# Neuronal MHC Class I Expression Is Regulated by Activity Driven Calcium Signaling

**DOI:** 10.1371/journal.pone.0135223

**Published:** 2015-08-11

**Authors:** Dan Lv, Yuqing Shen, Yaqin Peng, Jiane Liu, Fengqin Miao, Jianqiong Zhang

**Affiliations:** Key Laboratory of Developmental Genes and Human Disease, Ministry of Education, Department of Microbiology and Immunology, Medical School, Southeast University, Nanjing, Jiangsu Province, China; Stanford University School of Medicine, UNITED STATES

## Abstract

MHC class I (MHC-I) molecules are important components of the immune system. Recently MHC-I have been reported to also play important roles in brain development and synaptic plasticity. In this study, we examine the molecular mechanism(s) underlying activity-dependent MHC-I expression using hippocampal neurons. Here we report that neuronal expression level of MHC-I is dynamically regulated during hippocampal development after birth *in vivo*. Kainic acid (KA) treatment significantly increases the expression of MHC-I in cultured hippocampal neurons *in vitro*, suggesting that MHC-I expression is regulated by neuronal activity. In addition, KA stimulation decreased the expression of pre- and post-synaptic proteins. This down-regulation is prevented by addition of an MHC-I antibody to KA treated neurons. Further studies demonstrate that calcium-dependent protein kinase C (PKC) is important in relaying KA simulation activation signals to up-regulated MHC-I expression. This signaling cascade relies on activation of the MAPK pathway, which leads to increased phosphorylation of CREB and NF-κB p65 while also enhancing the expression of IRF-1. Together, these results suggest that expression of MHC-I in hippocampal neurons is driven by Ca^2+^ regulated activation of the MAPK signaling transduction cascade.

## Introduction

The major histocompatibility complex (MHC) is an extended cluster of genes, found in all jawed vertebrates encoding a number of proteins involved in immune responses. Among them, MHC class I (MHC-I) proteins are hetero-trimers composed of a transmembrane heavy chain, a non-covalently attached ß-2-microglobulin subunit (ß2m), and a short peptide comprising 8–15 amino acids derived from self or foreign proteins [1]. MHC-I molecules are ubiquitously expressed in somatic cells, although at different levels in different cell types [2].

The immune functions of MHC-I molecules are recognized, but their roles in the central nervous system (CNS) had been ignored for a long time. The CNS was recognized as “immune privileged”, because of the blunted or delayed adaptive immune responses within the brain. The underlying cause was thought to be due to lack of MHC-I expression by healthy neurons under normal conditions [3, 4]. However, a series of results from experimental animals show that MHC-I is expressed by healthy neurons in the postnatal central and peripheral nervous system [5–15]. It has been proven that neuronal MHC-I molecules play important roles in synaptic formation, remodeling and plasticity, which are significantly different from their immunological functions [6, 11, 12, 16]. Recently it has also been reported that classical MHC-I molecules were expressed by neurons during development of the human cerebellum and visual system [17, 18], as well as in adult brain [19, 20]. These studies began to unveil new functions for immune molecules in the CNS that may have important clinical implications.

The expression of MHC-I is dynamically regulated by developmental, tissue-specific, and hormonal/cytokine-mediated signals. Their expression level is mainly controlled transcriptionally by the interaction of tissue-specific transcription factors with cognate DNA sequence elements on the extended class I promoter. The DNA sequence elements that mediate class I regulation have been mapped to distinct domains [2, 21–24] and a variety of DNA-binding transcription factors have been identified that interact either directly or indirectly with those regulatory elements [25–28]. It has been reported that blockage of retinal activity by means of tetrodotoxin (TTX)-induced monocular deprivation of mice down regulates MHC-I expression levels in the LGN [6]. It has also been reported that MHC-I molecules are expressed in the developing visual cortex of the marmoset monkey and that their expression levels are regulated by neuronal activity [13]. However, the regulatory mechanism(s) underlying the activity-dependent expression of MHC-I by neurons has not been completely examined.

In the present study, we examined the regulation of MHC-I expression in the developing mouse hippocampus after birth and found that expression of MHC-I was dynamically regulated. In cultured mouse hippocampal neurons, increased neural activity driven by kainic acid (KA) stimulation drove significant enhancement of MHC-I expression. Three elements of the MHC-I promoter that bind to transcription factors NF-κB, IRF-1 and CREB played a vital role in KA induced expression of MHC-I genes. The phosphorylation of NF-κB p65, CREB and the expression level of IRF-1 were elevated by protein kinase C (PKC) upon KA treatment. Activation of PKC further activated the MAPK signaling cascade, thus activating transcription factors IRF-1, CREB and NF-κB, ultimately resulting in the increased expression of MHC-I after KA treatment. Taken together this report is the first to delineate an intracellular signaling pathway comprised of PKC, MAPK, CREB, NF-κB and IRF-1 for the up-regulation of MHC-I expression induced by KA in hippocampal neurons. Furthermore these findings suggest that activity dependent driven changes in Ca^2+^ may underlie temporal regulation of MHC-I expression in the hippocampus neurons.

## Materials and Methods

### Animals

All animals used in this study were C57BL/6J mice and maintained under pathogen-free conditions in our animal facility. All animal experiments were carried out in accordance with the protocols evaluated and approved by Institutional Animal Care and Use Committee (IACUC) of the Medical School of Southeast University (approval ID: SYXK-2010.4987).

### Cell lines and reagents

Neuro2a cells were purchased from the cell bank of the Type Culture Collection of the Chinese Academy of Science (Shanghai, China) and cultured in MEM (Gibco-BRL, Grand Island, NY, USA) supplemented with 10% fetal bovine serum (Gibco-BRL) and 1% penicillin-streptomycin antibiotics (Gibco-BRL). Kainic acid (KA), an agonist for ionotropic glutamate receptor, was purchased from Sigma-Aldrich (St. Louis, MO, USA) and dissolved in sodium citrate. Staurosporine (Beyotime Institute of Biotechnology, Shanghai, China), H89 (Beyotime), U0126 (Cell Signaling, Danvers, MA, USA) and Skepinone-L (ApexBio, Boston, MA, USA) were purchased and dissolved in dimethyl- sulfoxide (DMSO, Sigma-Aldrich).

### Hippocampal Cultures

Cultures were derived from postnatal day 0 to postnatal day 1 mouse hippocampus by using protocol reported previously [29]. Primary Hippocampal neurons were grown on six-well plates or on coverslips (coated with 100μg/mL poly-D-lysine) in 24-well plates for 7 to 9 days in Neurobasal-A media (Gibco-BRL) supplemented with B27 (Invitrogen, San Diego, CA, USA), GlutaMAX (Invitrogen), and D-glucose (Sigma–Aldrich). Half volume of the media was changed after 4 days *in vitro* (4 div). For neural activity manipulation, KA was added to 7 to 9 div cultures, vehicle control was sodium citrate (Sigma–Aldrich). Culture media was removed and replaced with drug/vehicle to a final concentration of 100μM for 30 minutes.

### Semiquantitative RT-PCR and RT-qPCR

Four male mice were investigated at each stage (P4, P8, P15, P30). Total RNA was extracted from mouse hippocampus, primary cultured hippocampal neurons, and Neuro2a using Trizol reagents (Invitrogen) according to the manufacturer’s instructions. Two micrograms of total RNA was treated with DNase I (Invitrogen) to eliminate contaminating genomic DNA. RNA was reverse transcribed using oligo(dT) primers and M-MLV (Promega, Madison, WI, USA) according to the manufacturer’s protocol. The expressions of genes were assessed by PCR using a Taq Master mix (Generay, Shanghai, China) or qPCR using SYBR Green PCR Master Mix (Applied Biosystems, San Diego, CA, USA). The primers used in this study were as follows: H-2K^b^, forward-5’-GCTGGTGAAGCAGAGAGACTCAG-3’, reverse-5’-GGTGACTTTATCTTCAGGTCTGCT-3’; H-2D^b^, forward-5’-AGTGGTGCTGCAGAGCATTACAA-3’, reverse-5’-GGTGACTTCACCTTTAGATCTGGG-3’; Tuj1 (βIII-tubulin), forward-5’-CGAGACCTACTGCATCGACA-3’, reverse-5’-CATTGAGCTGACCAGGGAAT-3’. The sizes of the PCR products were verified to be the predicted size by resolving on 1.5% agaorose gel electrophoresis and each PCR analysis was repeated at least three times. The RT-PCR bands of the target genes were quantified using an optical densitometer (SensiAnsys Gel Documentation and Analysis System, Shanghai, China) and normalized with the intensity of the bands of the internal control gene.

### Western blotting analysis

The whole-cell lysates were lysed by protein lysis buffer (50mM Tris-Cl PH7.4, 150mM NaCl, 1% NP-40, 0.5% Sodium deoxycholate, 0.1%SDS) with protease inhibitor and phosphatase inhibitor. Tissue samples (four male mice are used at each stage) from the hippocampus (P4, P8, P15, P30) were homogenized in protein lysis buffer with protease inhibitor and phosphatase inhibitor. Protein concentration was quantified by the Bradford assay (Pierce, Rockford, IL, USA) using BSA as the standard. Equal amounts of protein (20 μg) were denatured at 100°C for 5 min in a protein sample buffer, separated on a 10% SDS-PAGE and transferred to a polyvinylidene fluoride (PVDF) membrane (Millipore, Bedford, MA, USA). Membranes were incubated in 5% skimmed milk in tris-buffered saline with 0.05% Tween 20 (TBST buffer) at room temperature (RT) for 2 hours to block nonspecific binding, and then probed with primary antibodies overnight at 4°C. The primary antibodies and concentrations used were as follows: mouse anti-MHC-I (ox18, 1:1000; Abcam), rabbit anti-IRF-1 antibody (1:500; Santa Cruz, Dallas, TX, USA), rabbit anti-CREB and p-CREB (ser133) antibody (1:1000; Cell Signaling), rabbit anti-p65 and p-p65 (Ser536) antibody (1:1000; Cell Signaling), rabbit anti-JAK1 and p-JAK1 (Y1022) antibody (1:1000, Bioworld, St. Louis Park, MN, USA), rabbit anti-STAT1 and p-STAT1 (Tyr701) antibody (1:1000; Cell Signaling), rabbit anti-p38 and p-p38 antibody (1:1000; Cell Signaling), rabbit anti-ERK1/2 and p-ERK1/2 (Thr202/Tyr204) antibody (1:1000, Cell Signaling), rabbit anti-AKT and p-AKT (Ser473) antibody (1:1000; Cell Signaling), rabbit anti-synaptophysin antibody (1:1000, Santa Cruz), mouse anti-PSD-95 antibody (1:500, Thermo Fisher Scientific, Rockford, IL, USA), rabbit anti-c-fos antibody (1:500, Santa Cruz). The membrane was then washed using TBST and incubated with a horseradish peroxidase-conjugated secondary antibody for 1 hour at RT. Immunodetection was performed with an enhanced chemiluminescent (ECL) substrate (Pierce, Rockford, IL, USA). After ECL detection, the membranes were incubated in stripping buffer (62.5 mM Tris—HCl, pH 6.7; 2% SDS; 0.7% β- mercaptoethanol) at 60°C for 30 min, and then reprobed with rabbit anti-Tuj1 antibody (1:5000, Covance) or mouse anti-β-actin antibody (1:5000, Sigma) as a loading control for protein quantification. The signals were imaged with a luminoimage analyzer (Tanon, Shanghai, China), and quantification was performed by densitometry using Image J software (NIH).

### Immunofluorescence staining

Primary antibodies used in immunofluorescence experiments were: ER-Hr52 (which recognizes H-2K^b^ and H-2D^b^, the two classical MHC-I in C57BL/6 mice, 1:150, AbD Serotec, Oxford, UK), mouse anti-GFAP (1:1000, Chemicon, Bedford, MA), rabbit anti-synaptophysin antibody (1:200, Santa Cruz), mouse anti-PSD-95 antibody (1:200, Thermo Fisher Scientific).

Brains were fixed in 4% paraformaldehyde (wt/vol) in 0.01 M PBS overnight, cryo-protected in 30% sucrose at 4°C and then embedded in OCT. Coronal brain sections (20 μm) were rinsed in 0.01M PBS phosphate-buffered saline (PBS) for 5 min and permeabilized with PBT (PBS with 0.1%Triton X-100) for 30 min. Non-specific antibody binding sites were blocked with 10% normal serum in PBT for 2 hours at RT. Sections were then incubated overnight at 4°C in a humid chamber with primary antibodies. Washed 3 times with PBT, the sections were incubated with a secondary antibody for 3 h in a humid chamber at RT. After a final washing with PBS, the slides or coverslips were mounted with the mounting medium (Prolong Gold Anti-fade Reagent; Invitrogen, Grand Island, NY, USA). Incubations with the first antibody that substituted by isotype control antibody served as negative controls. The staining of spleen section was served as positive controls.

For primary hippocampal neurons’ protein labeling, coverslips were fixed by 4% paraformaldehyde (wt/vol) in 0.01 M PBS with 4% sucrose (wt/vol) for 20 min. Cells were washed with PBS for 5 min, permeabilized with 0.25% Triton X-100 (wt/vol) for 5 min and rewashed 3 times with PBS. Neurons were then blocked for 30 min with 10% bovine serum albumin (BSA, wt/vol) in PBS and incubated with primary antibodies overnight at 4°C in a humid chamber. After washing 3 times with PBS (with 3% BSA), coverslips were incubated with secondary antibodies for 1 hour. Following secondary binding, cells were rinsed with PBS and mounted on slides in Prolong Gold Anti-fade Reagent (Invitrogen).

The slides and coverslips were then observed under a laser scanning confocal microscope (Olympus Fluoview FV1000, Tokyo, Japan)

### Reporter plasmids and luciferase reporter assay

The pGL3.0-AP, pGL3.0-APD, pGL3.0-APD1, pGL3.0-APD2 and pGL3.0-APD3 reporter plasmids were generated by PCR and subsequently cloned as a 217-bp, 200-bp, 186-bp, 146-bp and 106-bp HLA-A*0201 promoter fragment upstream of the firefly luciferase gene in pGL3.0-Basic Vector (Promega). For the deletion of ISRE construct, the deleted base pairs were CGCAGTTTCTTTTCTCC, which span−185 to −169 of the HLA-A promoter. All the constructs were verified by DNA sequencing.

Neuro2a cells were seeded in a 24-well plate. Using the Lipofectamine 2000 (Invitrogen), the cells were co-transfected with the series of plasmids accompanied by pRL-SV40 (Promega), which served as a control to determine transfection efficiency. With or without 100μM KA stimulation for 30 min, the cells were lysed and luciferase activity was assayed with the Promega Dual Glo assay kit by using a TD 20/20n luminometer (Turner Biosystems, Sunnyvale, CA, USA). The data represented the average of three independent experiments and were shown with the standard error.

### Loading of cells with fluo-3 AM and fluorescence measurements

Changes in the levels of intracellular Ca2^+^ were measured as previously described [30]. Briefly, hippocampal neurons were plated in 35-mm glass bottom dishes for 8 days and were loaded with 4μM of the intracellular Ca2^+^-sensitive fluorescent dye Fluo-3 ⁄AM (Beyotine) at 37°C for 30 minutes. At the end of the incubation with Fluo-3 ⁄AM, cells were washed and incubated for an additional 30 minutes in a Fluo-3 ⁄AM–free Locke’s buffer to remove extracellular traces of the dye and to complete intracellular de-esterification. The fluorescence was then recorded for 10 min just after addition of KA using a laser scanning confocal microscope (Olympus) at 488 nm excitation and 538 nm emission wavelengths.

### Statistical analysis

Three or more independent experiments were performed. Data are presented as means±SD. The data were analyzed by Student's t-test or one-way anova. P < 0.05 was considered as statistically significant.

## Results

### Temporal expression of MHC-I during hippocampal development

We have previously reported that the expression of classical MHC-I molecules H-2K^b^ and H-2D^b^ mRNA varies from P0 (postnatal day 0) to P60 in mouse hippocampus, and that the dynamic expression of MHC-I molecules occurs during the development of human hippocampus [15, 31]. In this study, the distribution of H-2K^b^/D^b^ proteins was detected by immunofluorescent labeling. H-2K^b^/D^b^ proteins were found to be expressed by hippocampal neurons, but not by glial fibrillary acidic protein (GFAP) positive astrocytes (Figure A in S1 File). To examine the expression level of MHC-I molecules in neurons, quantitative analysis was performed on mouse hippocampus during development using RT-qPCR and western blotting.

We found that mRNA levels of MHC-I increased steadily from age P4 to P15, an important developmental period of mouse hippocampus (Figure B in S1 File). The change in MHC-I protein levels was well correlated with that in MHC-I mRNA before P15. However, after that, the expression of MHC-I protein declined while high levels of MHC-I mRNA remain expressed (Figures C and D in S1 File). These results indicate that expression of MHC-I by hippocampal cells is mainly controlled at the transcriptional level between ages P4 to P15 and that post-transcriptional regulation may play a role after P15.

### Expression levels of MHC-I molecules are regulated by neural activity *in vitro*

**Fig 1:**
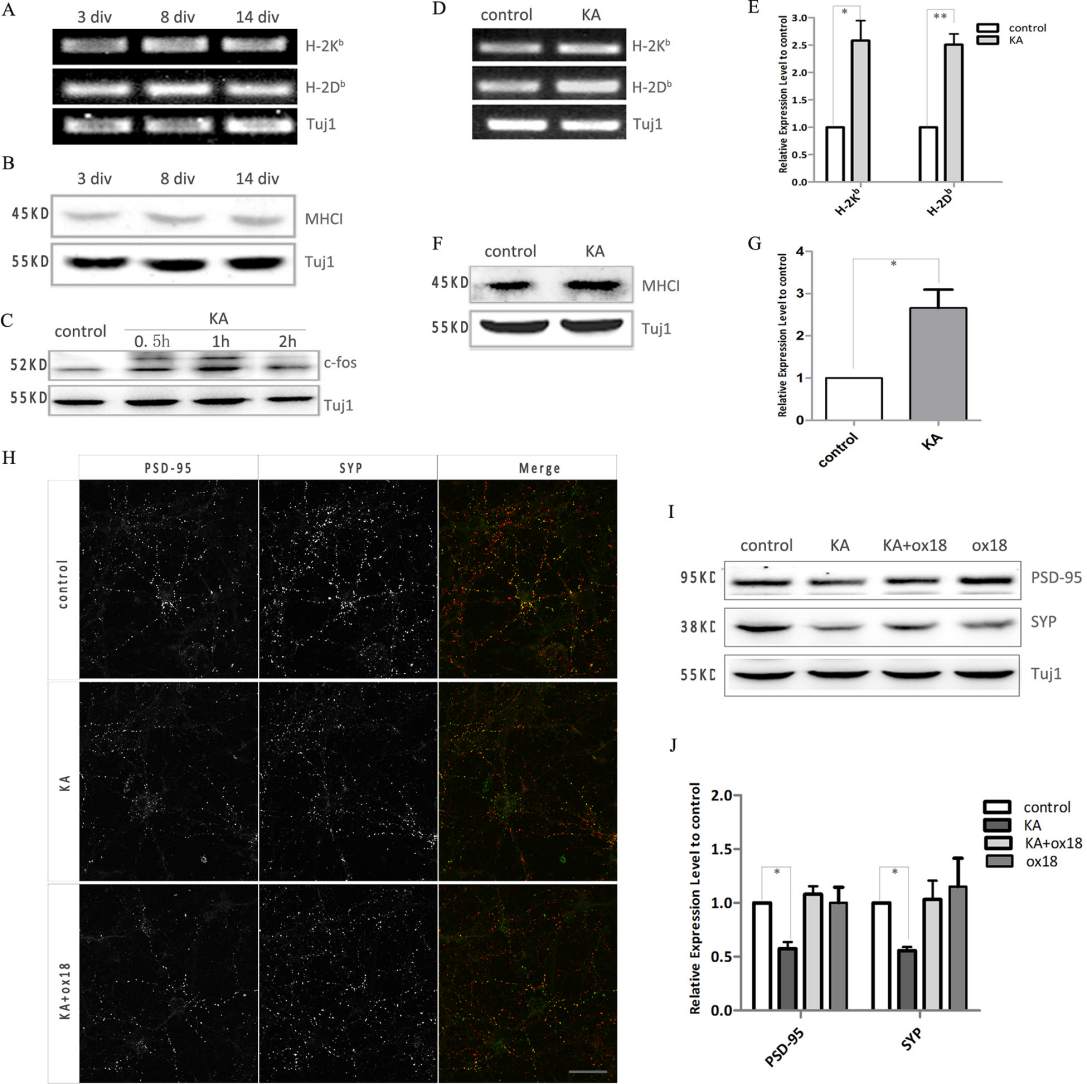
KA induced expression of MHC-I by hippocampal neurons play a role in the destabilization of synapse. Expression levels of MHC-I mRNA (A) and protein (B) were not varied during the development of cultured hippocampal neurons *in vitro*. (C) The expression of c-fos increased soon after adding 100μM KA to 8 div hippocampal neurons for 30 min. (D) Eight hours after adding 100μM KA to hippocampal neurons, the expression level of H-2K^b^ and H-2D^b^ mRNA significantly increased. (E) Expression of MHC-I mRNA was quantified as the ratio of band density to that of Tuj1. Data was presented as the ratio compared to control and was calculated from three independent experiments. (F) The expression level of MHC-I protein was increased significantly at 8 hours after KA treatment. (G) Expression of MHC-I protein was quantified as the ratio of band density to that of Tuj1. Data was presented as the ratio compared to control and was calculated from three independent experiments. (H) Forty-eight hours after 100μM KA stimulation for 30min, the expression of the pre-synaptic marker protein SYP (red) and post-synaptic marker protein PSD-95 (green) as well as their colocalization was decreased. The effect was partially rescued by using MHCI antibody ox18. Scale bar: 50 μm. (I) Western blot analysis showed increased expression of SYP and PSD-95 after KA stimulation, which was blocked by ox18. (J) Expression of SYP and PSD-95 was quantified as the ratio of band density to that of Tuj1. Data was presented as the ratio compared to control and was calculated from three independent experiments. *p<0.05, **p<0.01 vs control.

To further elucidate the mechanism(s) underlying regulation of MHC-I expression by hippocampal neurons, cultured hippocampal neurons were used as a cellular model *in vitro*. MHC-I mRNAs and proteins were detected in neurons 3 days *in vitro* (3 div) before synaptogenesis, as well as 8 div and 14 div, during the synaptogenesis of hippocampal neurons. To our surprise, the expression level of endogenous MHC-I did not vary with age in cultured neurons (Fig 1A and 1B).

It is known that synaptic input regulates gene expression in target neurons and previous studies have reported that higher expression levels of MHC-I in the visual cortex were associated with increased retinal activity [6, 13]. However, whether the expression of MHC-I in hippocampal neurons is regulated by neural activity has remained ambiguous [3, 12, 32]. Kainic acid (KA) stimulates neurons by binding to glutamic kainate receptors. We measured the change of MHC-I expression following KA stimulation in cultured hippocampal neurons during the synaptogenesis (8 div). One hour after KA stimulation, the expression of c-fos, a marker of neuronal activity was increased in hippocampal neurons (Fig 1C). Increased MHC-I expression was detected at 8 hours after KA stimulation, and the change of protein levels correlated well with the level of mRNA (Fig 1D–1G), implying regulation at the level of transcription for induced MHC-I expression. Furthermore, western blot analysis showed the expression of both the pre-synaptic protein synaptophysin (SYP) and post-synaptic protein PSD-95 decreased 48 hours after KA stimulation. In accordance with this, KA stimulation produced a reduction in staining and co-localization of pre- and post-synaptic protein clusters. Pre-treatment with the anti-MHC-I antibody “ox18” (1.0 μg/mL) partially rescued hippocampal neurons from the KA-induced down-regulation of pre- and post-synaptic proteins (Fig 1H–1J). Taken together these results indicate that stimulation of hippocampal neurons with KA upreglates MHC-I expression which may in turn participate in the down regulation of synaptic densities.

### NF-κB, CREB and IRF-1 participate in the regulation of MHC-I expression in hippocampal neurons

**Fig 2:**
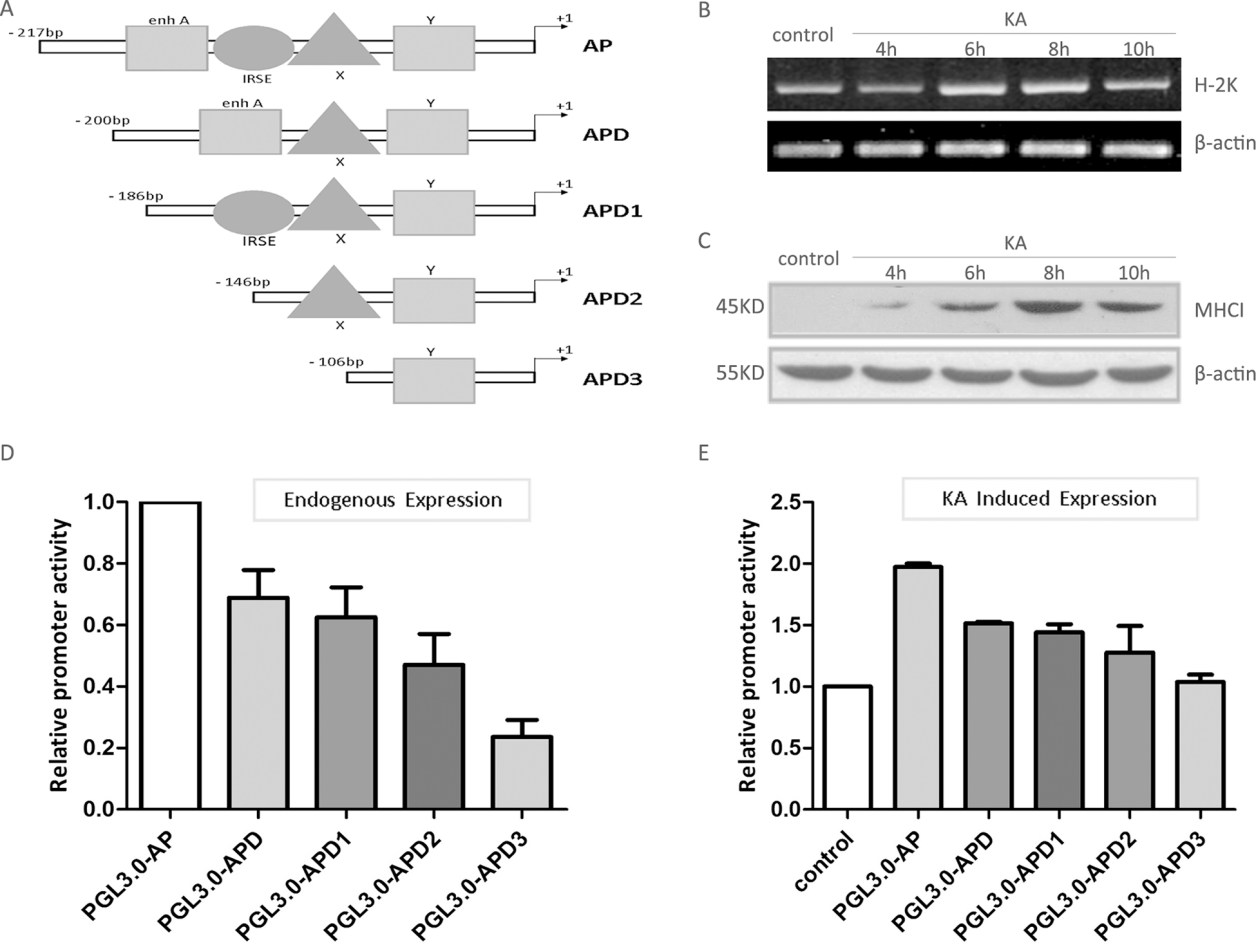
Enh A, ISRE and X-box are important elements for the endogenous and KA induced MHC-I promoter activity in neuro2a cell lines. (A) Schematic illustration of constructs used for HLA-A heavy chain promoter-luciferase reporter assay. (B) At 6, 8 and 10 hours post 100 μM KA treatment for 30min increased the expression level of H-2K mRNA and protein (C). (D) Luciferase activity of different promoter-reporter plasmids in neuro2a cell lines. Mean luciferase activity was calculated from three independent experiments and shown with the SD. (E) Relative luciferase activity of different promoter-reporter plasmids in neuro2a cell lines by using 100μM KA for 30min.

Three major regulatory elements exist in the MHC class I promoter: the enhancer A (enh A), interferon stimulated response element (ISRE), the site-α and enhancer B (also named SXY module) [23, 33]. A variety of transcription factors bind directly or indirectly to these elements in different cells to mediate diverse routes of endogenous and induced transcription of MHC-I genes. In order to clarify the role of these regulatory promoter elements and their corresponding transcription factors in the expression of MHC-I in neurons, a series of truncated MHC-I promoter luciferase reporter plasmids were transfected into Neuro2a cells, which also showed an increase in MHC-I expression upon KA stimulation (Fig 2A–2C). The results showed that deletion of enh A or/and IRSE resulted in a reduction of MHC-I promoter activity. Truncation of enh A, IRSE and siteα elements led to a marked decrease in gene transcription (Fig 2D). Meanwhile, KA induced activation of MHC-I promoter was also influenced by depletion of these elements. As predicted, the induction of luciferase activity by KA was almost entirely abolished with deletion of all the three elements: enh A, IRSE and siteα (Fig 2E). These results indicate that all of these three elements play a role in endogenous and KA induced expression of MHC-I genes.

**Fig 3:**
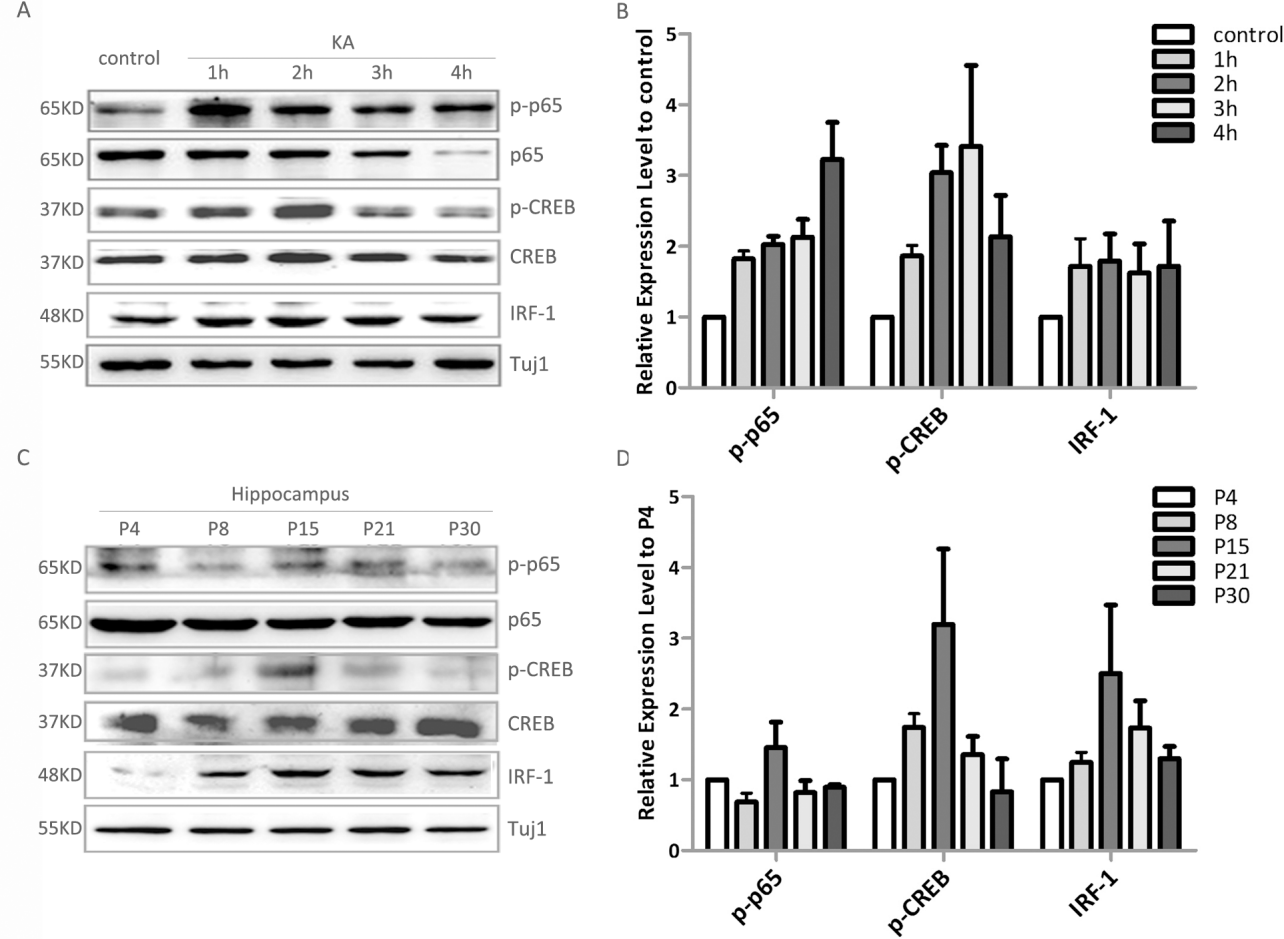
Involvement of NF-κB, CREB and IRF-1 in neuronal MHC-I expression. (A) Western blot analysis of time-dependent activation of NF-κB p65, CREB as well as the increased expression of IRF-1 by adding 100μM KA to 8 div hippocampal neurons for 30min. (B) The densitometric analyses of p-p65/p65, p-CREB/CREB, and IRF-1/Tuj1 from three separate experiments were taken, and the data was shown as ratio compared to control. All the data are indicated as mean±SD. (C) Western blot analysis of the expression level of of NF-κB p65, CREB as well as the expression of IRF-1 during the development stages of mouse hippocampus. (D) The densitometric analyses of p-p65/p65, p-CREB/CREB, and IRF-1/Tuj1 from four separate experiments were taken, and the data was shown as ratio compared to P4. All the data are indicated as mean±SD.

Enhancer A, ISRE and siteα are the binding sites for nuclear transcription factors of the NF-kB/Rel family, IRF family and CREB, respectively. We next investigated the state of these transcription factors with or without KA stimulation. Results showed that the phosphorylated form of NF-κB p65 (p-p65) and CREB (p-CREB) were increased after KA treatment, while the expression of the IRSE-binding factor IRF-1 was increased rapidly to a steady state level (Fig 3A and 3B). The tight-correlation of increased transcription factors, activation of the MHC-I promoter and expression of MHC-I protein demonstrates the importance of these transcription activity of these factors in the regulation of MHC-I expression following KA stimulation. Furthermore, the dynamic expression of p-p65, p-CREB and IRF-1 were all well correlated with that of MHC-I during the development of mouse hippocampus (Fig 3C and 3D), suggesting that they might participate in the developmentally regulated expression of MHC-I by hippocampal neurons *in vivo*.

### Ca^2+^-dependent PKC and protein kinase A (PKA) are involved in KA induced expression of MHC-I by hippocampal neurons

**Fig 4:**
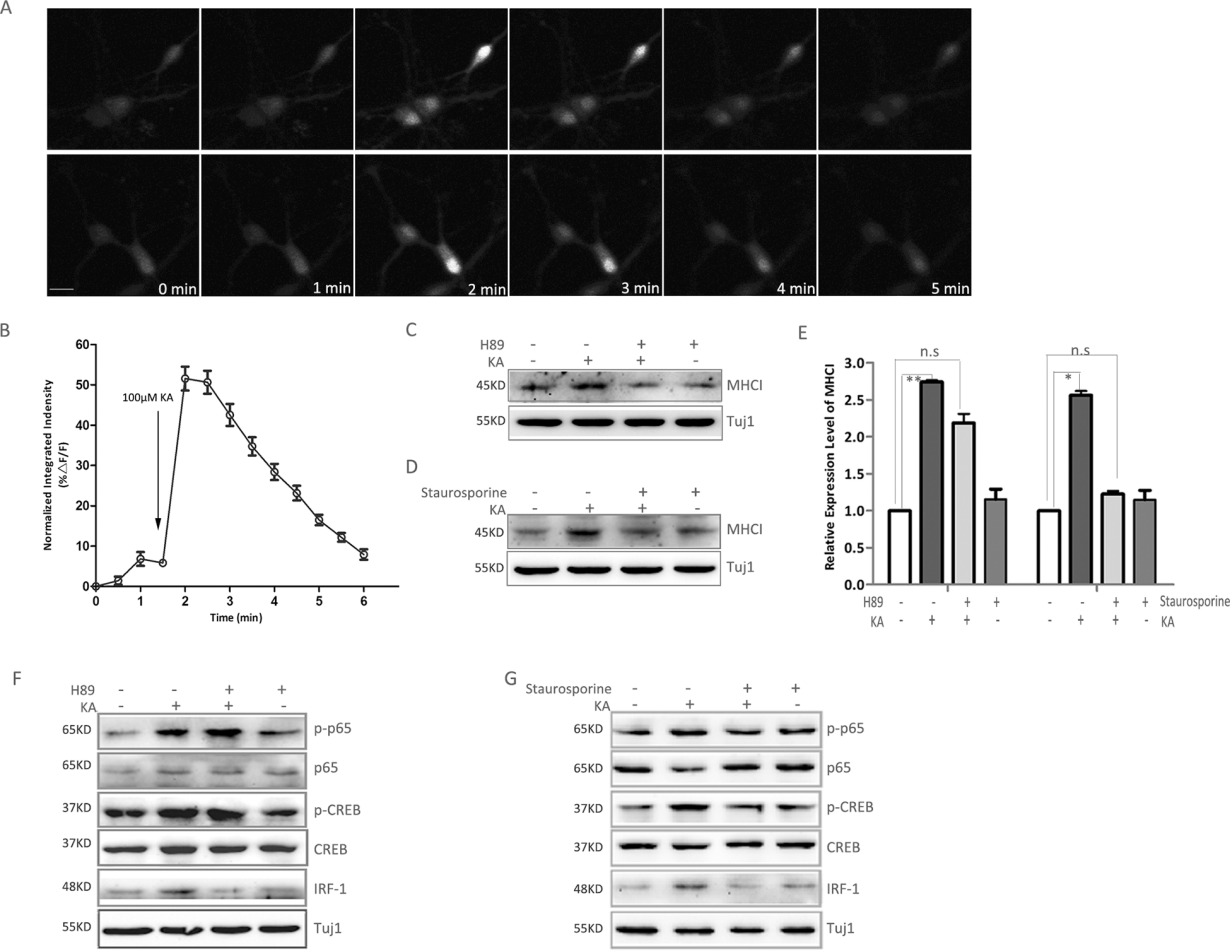
Calcium-dependent PKA and PKC activation participate in KA induced MHC-I expression. (A) Two representative cells showed the increased concentration of intracellular calcium soon after adding 100μM KA. Scale bar: 50 μm. (B) Average normalized fluorescence intensity before and after KA (100μM) treatment. Each bar represents the mean ± SD of 50 cells. (C) Pretreatment of primary hippocampal neurons with PKA inhibitor (H89, 40μM) resulted in inhibition of KA-induced expression of MHCI. (D) Pretreatment of primary hippocampal neurons with PKC inhibitor (Staurosporine, 0.1μM) blocked KA-induced expression of MHC-I. (E) Densitometric analyses of MHC-I/Tuj1 at 8 hours after KA treatment from three separate experiments were taken, and the data was shown as ratio compared to control. All the data are indicated as mean±SD. (F) Pretreatment of primary hippocampal neurons with PKA inhibitor (H89, 40μM) resulted in inhibition of KA-induced expression of IRF-1, but had no effect on the activation of NF-κB p65 and CREB. (G) Pretreatment of primary hippocampal neurons with PKC inhibitor (Staurosporine, 0.1μM) blocked KA-induced expression of p-p65, p-CREB and IRF-1. n.s: no significance, *p<0.05, **p<0.01 vs control.

We next set out to examine the signaling pathways leading to KA induced activation of MHC-I transcription factors. Similar to KA stimulation, stimulation of glutamate receptors causes membrane depolarization which opens Ca^2+^ channels, resulting in a rise in the intracellular Ca^2+^ concentration [34]. We hypothesized that Ca^2+^ may play an important role in the transcriptional regulation of MHC-I expression. To test this hypothesis, we measured intracellular Ca^2+^ using a Ca^2+^-sensitive fluorescent dye to detect a sharp increase of intracellular Ca^2+^ concentration upon KA stimulation. As shown in Fig 4A and 4B, the concentration of Ca^2+^ rose to peak at 30 seconds after KA treatment before returning quickly to the baseline level. The results show that KA induced a transient increase of intracellular Ca^2+^ in cultured neurons.

Since PKA and PKC play a pivotal role in the Ca^2+^ signaling pathways, and are able to trigger the activity of many kinases following binding with calcium, we explored the possibility that these two kinases could be involved in KA induced MHC-I expression. Thus, we examined whether inhibitors of PKA and PKC were able to block KA induced MHCI expression. After the treatment with H89 (an inhibitor of PKA) or staurosporine (an inhibitor of PKC), the increased expression of MHC-I by KA was compromised (Fig 4C–4E). Furthermore, the KA induced activation of NF-κB p65 and CREB was diminished by using staurosporine, while the increased expression of IRF-1 was inhibited either by H89 or staurosporine (Fig 4F and 4G). These results suggest that a calcium-dependent PKC and PKA pathways are activated by KA stimulation. The PKC and PKA pathway may therefore serve as the upstream signal for the activation of NF-κB p65, CREB and IRF-1, and finally increase the expression of MHC-I.

### PKC mediated activation of MAPK pathways participates in MHC-I expression induced by KA stimulation

**Fig 5:**
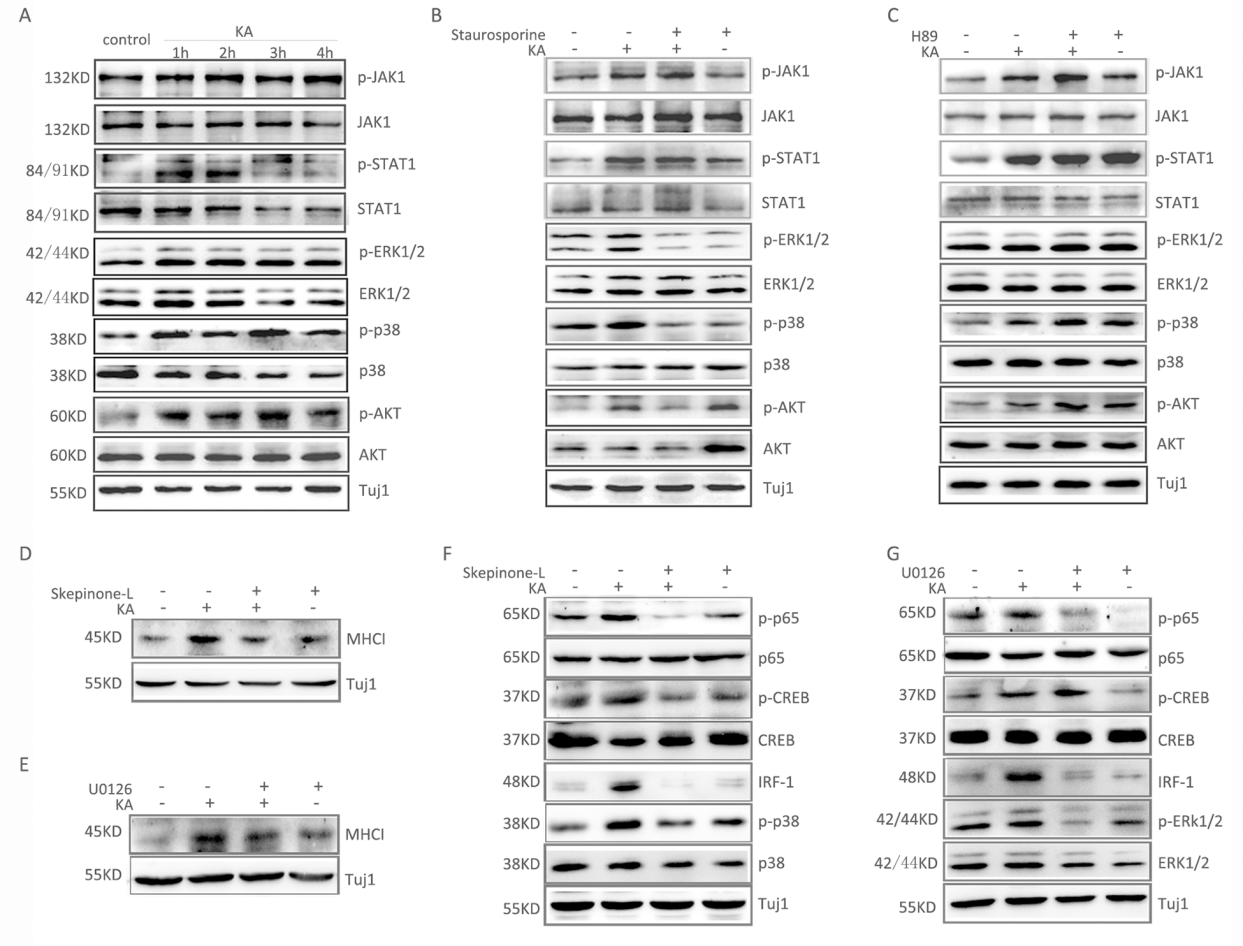
PKC mediated activation of MAPK and AKT pathways by KA stimulation. (A) Western blot analysis of time-dependent activation of JAK1, STAT1, AKT, MAPK ERK1/2 and p38 after adding 100μM KA to 8 div hippocampal neurons for 30min. (B) Pretreatment of 8 div hippocampal neurons with PKC inhibitor (Staurosporine, 0.1μM) compromised the KA-induced activation of AKT, MAPK ERK1/2 and p38, but had no effect on the activation of JAK1 and STAT1. (C) Pretreatment of 8 div hippocampal neurons with PKA inhibitor (H89, 40μM) had no effect on the activation of JAK1, STAT1, AKT, MAPK ERK1/2 and p38 induced by KA. (D) Pretreatment of primary hippocampal neurons with MAPK-p38 inhibitor (Skepinone-L, 20μM) resulted in inhibition of KA-induced expression of MHC-I. (E) Pretreatment of primary hippocampal neurons with MAPK-ERK inhibitor (U0126, 10μM) blocked KA-induced expression of MHC-I. (F) Pretreatment of primary hippocampal neurons with MAPK-p38 inhibitor (Skepinone-L, 20μM) blocked KA-induced expression of p-p65, p-CREB and IRF-1. (G) Pretreatment of primary hippocampal neurons with MAPK-ERK inhibitor (U0126, 10μM) resulted in inhibition of KA-induced expression of IRF-1 and activation of NF-κB p65, but had no effect on the activation of CREB.

We next examined some signaling pathways that have been reported as upstream signals of MHC-I’s three transcription factors to see if they are activated upon KA treatment. Results showed that the JAK1/STAT1, AKT, MAPK/ERK and MAPK/P38 pathways were all activated after KA stimulation (Fig 5A). Pre-treatment with staurosporine, but not H89, could block the phosphorylation of ERK, P38 and AKT induced by KA (Fig 5B and 5C). This implies KA induced activation of these three pathways is mediated by PKC. However, phosphorylation of JAK1 and STAT1 was not affected either by staurosporine or H89, indicating their response to KA stimulation is not mediated by activation of PKA or PKC.

Next, we examined whether inhibitors of ERK and p38 were able to block KA induced MHC-I expression. Pre-treatment with U0126 (an inhibitor for MAPK/ERK) and Skepinone-L (an inhibitor for MAPK/P38) blocked KA induced expression of MHC-I (Fig 5D and 5E). Further detection demonstrated that U0126 could compromise the phosphorylation of NF-κB p65 and the expression of IRF-1, while Skepinone-L could block the activation of NF-κB p65, CREB and IRF-1 upon KA treatment (Fig 5F and 5G).

## Discussion

**Fig 6:**
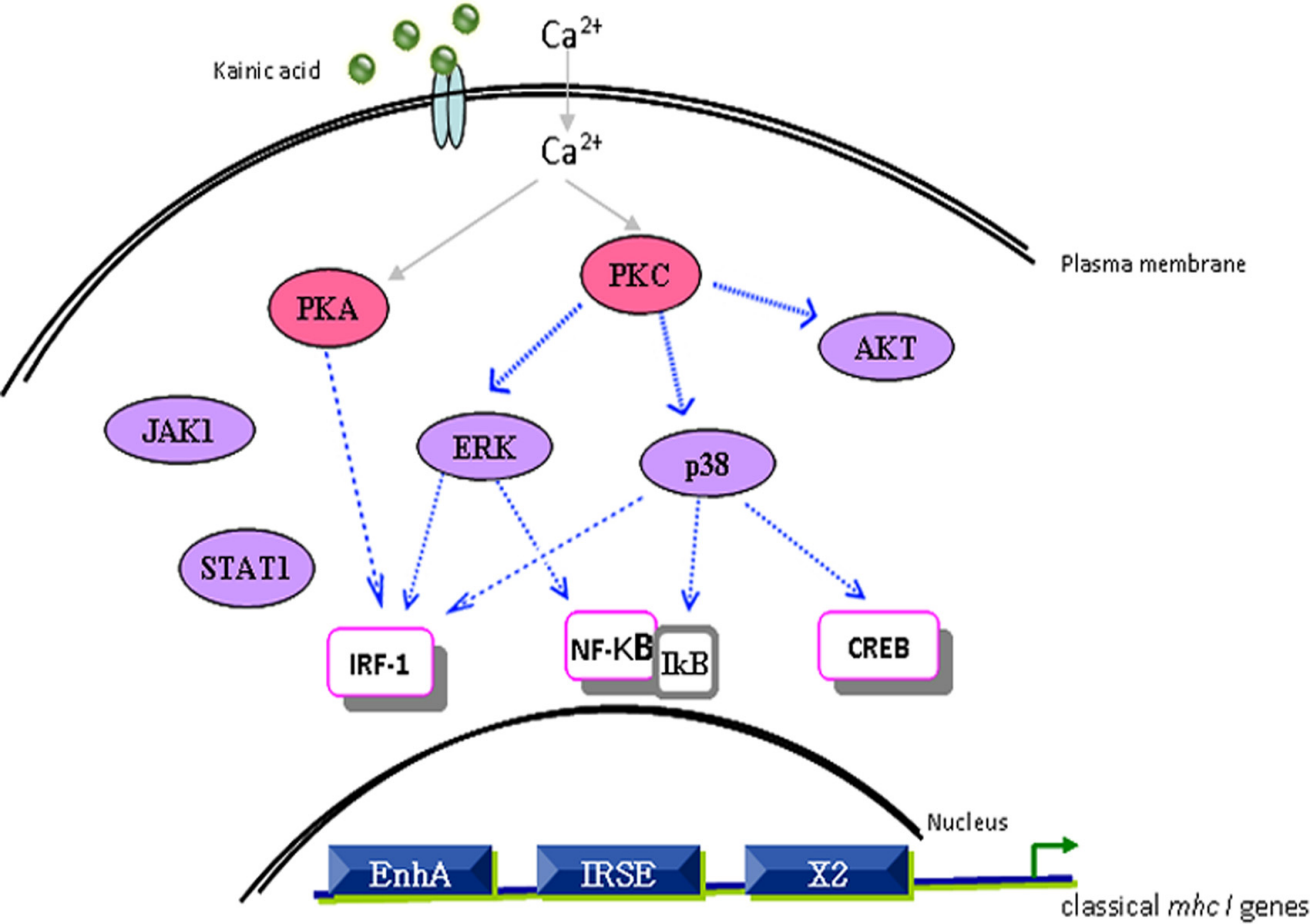
Model depicting the calcium-dependent pathway mediated MHC-I expression induced by KA. Exposure of hippocampal neurons to KA leads to activation of calcium-dependent PKA and PKC, which results in the subsequent activation of the MAPK pathways and the downstream transcription factors NF-κB, CREB and IRF-1. Activation of these molecules finally leads to enhanced expression of MHC-I by binding to its promoter elements. JAK1/STAT1 and AKT pathways are also activated by KA stimulation.

In this study, we examined mechanisms underlying the regulated expression of MHC-I by hippocampal neurons. First we identified *in vivo* developmentally regulated expression in mouse hippocampus and secondly found that KA stimulation induced expression of MHC-I in cultured hippocampal neurons. Transcription factors IRF-1, CREB and NF-κB, which has been proved to be able to combine to the MHC-I promoter in lymphocytes [25, 26, 35], participated in KA induced MHC-I expression in hippocampal neurons. KA-stimulation of hippocampal neurons initiated a Ca^2+^-dependent PKC mediated MAPK cascade which activated transcription factors IRF-1, CREB and NF-κB and ultimately resulted in increased expression of MHC-I. Taken together, we propose a model wherein Ca^2+^-dependent protein kinase C and its down-stream signaling pathways mediated neuronal activity induced MHC-I expression in hippocampal neurons (Fig 6).

In the immune system, MHC class I expression is mainly controlled transcriptionally by the interaction of tissue-specific transcription factors with cognate DNA sequence elements on the extended class I promoter [22]. It has been reported that three major regulatory promoter elements, Enhancer A, ISRE, and the SXY module mediated different routes of MHC class I genes transcription [25]. A variety of DNA-binding transcription factors have been identified that interact either directly or indirectly with tissue-specific and hormone/cytokine-specific regulatory elements. For example, a B lymphocyte–specific enhanceosome consisting of the coactivator CIITA and DNA-bound transcription factors RFX, CREB/ ATF, and NF-Y leads to high cell surface class I and II expression in B cells [36]. In T cells, the constitutive high level expression of class I is not due to CIITA but is established by a T cell enhanceosome consisting of RUNX1, CBFb, and LEF1 [37]. In this study, we demonstrated that MHC-I expression by hippocampal neurons in the central nervous system was also transcriptionally controlled. We find that a “neuronal specific enhanceosome” consisting at least of the NF-κB, IRF-1 and CREB, which binds to enhancer A, IRSE and X box respectively, participate in the endogenous and KA induced expression of MHC-I. However, neuroblastoma cells are substantially different from primary neurons. The use of cells in this study is just convenient for transfection experiments. Further studies are needed to investigate if these transcriptional factors work synergistically or independently and if they are also functional in primary cultured neurons. Based on our results, the immune system and central nervous system may take the same route to regulate MHC-I expression, although the stimulation signals might be different. In addition, some reports have shown that high levels of MeCP2, a transcriptional repressor that binds to methylated CpG di-nucleotides, can depress MHC-I expression in neuronal cells [38]. We cannot exclude the possibility that DNA methylation also plays a role in KA induced expression of neuronal MHC-I.

Stimulation of glutamate receptors leads to membrane depolarization which opens Ca^2+^ channels and result in a rise in the intracellular Ca^2+^ concentration [34]. This is consistent with our results that KA stimulation, an agonist of glutamate receptors, can increase intracellular Ca^2+^ concentration of hippocampal neurons. Calcium is an important second messenger which can activate downstream signals through binding with Ca^2+^-dependent protein kinases and Ca^2+^/calmodulin (Ca^2+^/CaM) dependent protein kinases and phosphatases [34, 39]. The phosphorylation of PKA and PKC is a key component of calcium-mediated signaling machinery. By using specific inhibitors, we found that increased level of intracellular Ca^2+^, accompanied by the downstream signaling through pathways including PKA and PKC, might be involved in the process of KA induced MHC-I expression. However, the roles of Ca^2+^/CaM-dependent protein kinases in mediating KA induced MHC-I expression are still unknown. We conclude that PKC serves as the upstream signal of NF-κB p65, CREB and IRF-1 since an inhibitor of PKC inhibited their activation. PKC could subsequently phosphorylate AKT and MAPK, thus activating the downstream signaling pathways. Although both PKA and PKC played a role in KA induced upregulation of IRF-1, phosphorylation of JAK1/STAT1, a known regulator of IRF-1, was not affected by inhibition of PKA or PKC. Other signaling pathway(s) might be responsible for activation of PKA and PKC cascades that result in IRF-1 up-regulation.

In conclusion, we find that expression of MHC-I by hippocampal neurons is regulated by a Ca^2+^ dependent transduction cascade in response to KA stimulation. We also observed KA induced expression of MHC-I can decrease the expression of pre-synaptic and post-synaptic proteins, which may finally influence the density of the glutamatergic synapses. It has been reported that MHC-I molecules are present in the plasma membrane of both axons and dendrites of cortical neurons before and during synapse formation and that they negatively regulate the density and function of glutamatergic connections [12]. In addition, neural activity has been reported to regulate glutamatergic synapse density in part through MHC-I [12]. In accordance with previous reports, the current results raise the possibility that physiological activity associated changes in neuronal MHC-I levels could decrease the number and the connectivity of synapses during CNS development. In addition, this possibility implies that under some pathological conditions, such as epilepsy, dysregulation of neuronal activity might alter the level of neuronal MHC-I, thus producing changes in synaptic connections and neuronal electrophysiology. MHC class I genes have been linked to a number of CNS disorders. Discovery of mechanism regulating MHC-I expression in neurons has shed new light on manipulation of their expression in these diseases [19, 40, 41].

## Supporting Information

S1 File Dynamic expression of H-2K^b^/D^b^ in mouse hippocampus. (A) At P15, MHCI protein signals (green) were overlapped with those of NeuN (red, a marker for neurons) but not with GFAP (red, a marker for astrocytes). Scale bar: 50 μm. (B) The mRNA of H-2K^b^ and H-2D^b^ was dynamically expressed in mouse hippocampus. Data was shown as ratio compared with P4 and was calculated from three independent experiments. (C) Western blot analysis depicting the expression of MHC-I protein during the development stages of hippocampus. (D) Expression of MHC-I protein was quantified as the ratio of band density to that of Tuj1. Data was presented as ratio compared with P4 and was calculated from three independent experiments.
(TIF)
