# Supplementary figures and images for: Neuronal MHC Class I Expression Is Regulated by Activity Driven Calcium Signaling
Source: PLoS One. 2015 Aug 11;10(8):e0135223. doi: 10.1371/journal.pone.0135223 (PMC4532511; doi:10.1371/journal.pone.0135223)

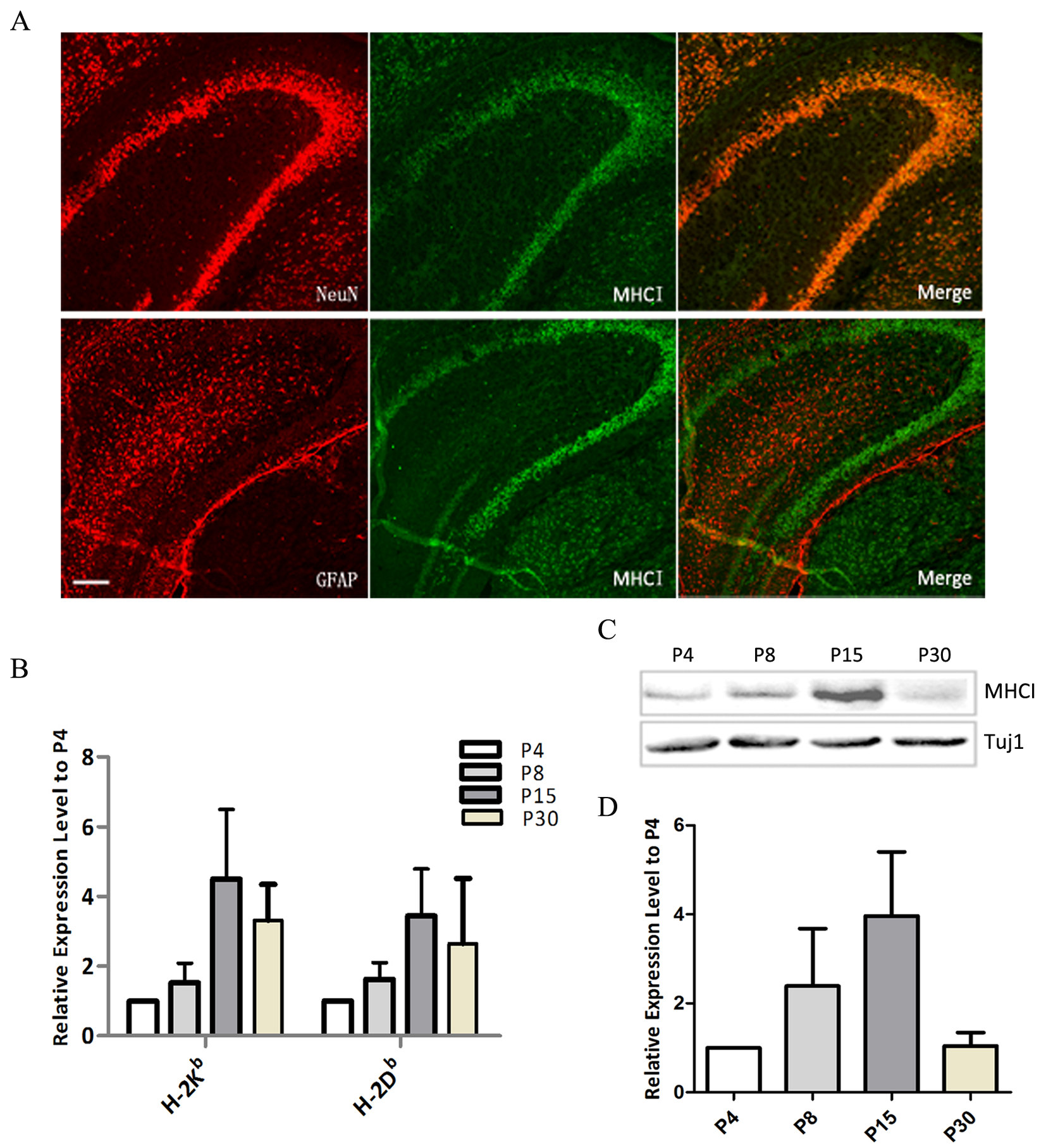

Supplement: S1 File — (A) At P15, MHCI protein signals (green) were overlapped with those of NeuN (red, a marker for neurons) but not with GFAP (red, a marker for astrocytes). Scale bar: 50 μm. (B) The mRNA of H-2Kb and H-2Db was dynamically expressed in mouse hippocampus. Data was shown as ratio compared with P4 and was calculated from three independent experiments. (C) Western blot analysis depicting the expression of MHC-I protein during the development stages of hippocampus. (D) Expression of MHC-I protein was quantified as the ratio of band density to that of Tuj1. Data was presented as ratio compared with P4 and was calculated from three independent experiments. (TIF) [file pone.0135223.s001.tif]
